# Force-time curve features of handgrip strength in fibromyalgia syndrome

**DOI:** 10.1038/s41598-020-60227-8

**Published:** 2020-02-25

**Authors:** Fausto Salaffi, Sonia Farah, Marco Di Carlo

**Affiliations:** Rheumatological Clinic, Ospedale “Carlo Urbani”, Università Politecnica delle Marche, Jesi (Ancona), Italy

**Keywords:** Skeletal muscle, Fibromyalgia

## Abstract

The aim of this study was to compare the handgrip strength (HGs), assessed with a cylindrical-shape grip device, of fibromyalgia syndrome (FM) patients with healthy subjects and to demonstrate the relationship between HGs characteristics and disease severity. Consecutive female patients with FM were enrolled and compared to a group of healthy women. The correlations between HGs curve characteristics and disease severity indices were studied through the Spearman’s rho correlation coefficients (rho). The HGs threshold distinguishing the FM presence was determined using the receiver operating characteristic (ROC) curves analysis. Multivariate regression analysis was used in order to assess the contribution of covariates on the HGs. 110 patients (mean age 53.8 ± 12.4 years) and 111 healthy controls have been enrolled. Altogether all parameters related to the analysis of HGs were worse in patients with FM. The HGs cut-off distinguishing the presence of a FM was 14.2 Kg. A negative correlation was found between disease severity indices and peak force (p < 0.001). Factors significantly associated with HGs area under the curve (AUC) in multivariate analysis were the Widespread Pain Index (WPI) (p = 0.003) and the revised Fibromyalgia Impact Questionnaire (FIQR) (p = 0.016). HGs is reduced in female FM patients and is inversely related to disease severity. The force-time curves analysis may be used as a complementary tool in the FM assessment and monitoring.

## Introduction

Fibromyalgia syndrome (FM) is a multi-symptom disorder, characterized mainly by chronic widespread musculoskeletal pain, chronic fatigue, sleep disturbance, symptoms of cognitive impairment, and activity limitations, the etiology of which is still to be clarified^1^.

The physical fitness is globally reduced over the course of FM and under this term are included multiple domains, in particular muscle strength, aerobic activity, balance and flexibility: the limitations of one or more of these aspects determine a functional limitation^2,3^. Adequate muscle strength is obviously necessary to perform all the usual actions of daily life^4–6^. In female patients with FM, a strength deficit of 20% to 36% is documented compared to healthy women^7^. The reduction in muscle strength in women with FM results in a deterioration in the quality of life (QoL)^8^.

Handgrip strength (HGs), as a substitute for overall muscle strength, is related to physical fitness^9,10^. HGs is traditionally measured as the maximum force that can be generated by each hand. This parameter is used to assess the degree of hand deterioration in the FM. However, peak force measurement alone does not take into account other hand strength characteristics such as force generation speed, sustainability, and grip force variability.

A more accurate description of HGs is certainly provided by the force-time (FeT) curve analysis. The FeT curve can be used to assess effort sincerity and distinguish between sincere effort and false weakness^11^. Through the analysis of the FeT curve it is possible to reveal peak force, mean force, total grip time, area under the curve, and variability of the curve plateau region^12^.

To the best of our knowledge, such kind of assessment has never been conducted in FM patients.

On the basis of these considerations, the objective of this study was to explore the FeT curve analysis of HGs, using a cylindrical-shape grip device, in patients suffering from FM compared to healty subjects.

## Methods

### Subjects

From July 2018 to May 2019, consecutive adult FM patients of the Rheumatological Clinic of the Università Politecnica delle Marche (Jesi, Ancona, Italy), fulfilling the 2016 American College of Rheumatology (ACR) classification criteria for FM (using the cut-off of 13 to classificate FM according the Symptom Severity Score) were enrolled in this study^13,14^. Only female patients were included for the purposes of this study.

All the patients underwent the rheumatologic assessment to confirm the FM diagnosis at study entry. Exclusion criteria were represented by the presence of: (a) coexisting inflammatory rheumatic diseases or connective tissue disorders, (b) orthopedic or musculoskeletal conditions that would prohibit moderate-intensity exercise (i.e. severe osteoarthritis); (c) known cardiovascular disease or uncontrolled hypertension; (d) moderate-severe chronic lung disease; (d) uncontrolled endocrine disturbances; (g) history of major depression disorder, schizophrenia or other psychosis.

Each patients underwent a comprehensive clinimetric evaluation including socio-demographic variables, disease duration, QoL items, and disease-related variables was administered to the patients.

The socio-demographic variables were: age, weight, height, marital status (single, married and divorced/separated), and level of education according to Italian school system, primary (from 6 to 10 years old); secondary (from 11 to 13 years old); high school/university (from 14 to 23 years old). FM severity was evaluated through two Italian validated patient-reported outcomes (PROs) vesions, designed for the multidimensional assessment of the disease: the revised Fibromyalgia Impact Questionnaire (FIQR) and the Fibromyalgia Assessment Status (FAS)^15,16^. The FIQR is a multidimensional tool widely disseminated to assess the impact of disease. It consists of 21 numerical rating scales (NRS) aimed at exploring function, overall impact, and symptoms during the last week^17,18^. The final score ranges from 0 to 100. The severity of the FM has been established on the basis of the FIQR score, in particular remission: FIQR ≤ 30, mild severity: 30 <FIQR ≤ 45, moderate severity: 45 <FIQR ≤ 65, and high severity: FIQR > 65^19^.

The FAS combines questions addressing fatigue, quality of sleep, and non-articular pain assessed by the Self-Assessment Pain Scale (SAPS)^16^. The final score ranges from 0 to 48, converted into a 0–10 scale.

For each participant was also registered the Patient Acceptable Symptoms State (PASS)^20^. PASS was recorded as “yes” or “no” answering to the anchor question: “Considering all the different ways your disease is affecting you, if you were to stay in this state for the next few months, do you consider that your current state is satisfactory?”^21^.

The present study was conducted in accordance with the 1964 Declaration of Helsinki principles and its later amendments or comparable ethical standards, and according to the rules of the Good Clinical Practice. The study design was approved by the the institutional review Board of the Hospital Clinic ethics committee (Comitato Etico Unico Regionale, prot. AV2 n° 22418) and informed written consent was provided by all participants.

### Handgrip strength measurement

**Figure 1:**
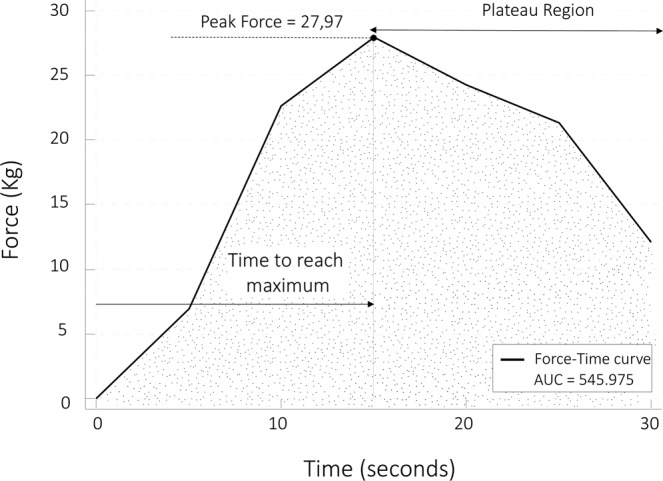
Force–time (FeT) curve showing the method of calculation of the various force attributes.

The HGs was assessed using a cylindrical-shape grip device made of five force sensors (FSR-402) manufactured by Interlink Electronics and connected to an Arduino Mega 2560. The device used in this study allowed the measurement of grip force in a time frame of 30 seconds, making one measurement per sensor every five seconds. Through these repeated measurements, it was possible to trace the FeT curves. A graphical representation of a typical FeT curve is provided in Fig. 1. In this study we used the peak grip force and the area under the curve force as the main outcomes, and time to reach maximum plateau of the curves as a secondary outcome. The HGs was measured in the dominant hand twice, and the mean of the two measurement values was used. The subjects were tested while sitting in a standardized position with elbow flexed at an angle of 90°.

The HGs features were also determined in a control group (111 patients) consisting of voluntary healthy women, properly comparable in terms of age (55.2 ± 14.9 years) and BMI, identified among the staff of the Rheumatological Clinic and in the relatives of the patients.

### Statistical analysis

The X–Y coordinates generated were transferred to a spreadsheet (Microsoft Excel 2011 for Mac, version 14.7.7) to generate digital curves, which were further processed with the MedCalc Statistical Software, version 18.0 (Ostend, Belgium), for Windows XP. Several parameters were assessed for each FeT curve. Peak force was the maximum grip strength achieved on the FeT curve, and the and variability of the time to reach maximum plateau of the curve was calculated from readings in the middle half of the force–time curve excluding the first and last quartiles, as this represents the force generation and relaxation segments. Total grip time was calculated using the X-axis of the FeT curve. The serial measurement method (first observation taken as baseline value of 0) was used in MedCalc to calculate the total area under the curve of HGs (HGs-AUC).

Median and interquartile ranges, as well as means and standard deviations (SDs) were presented where appropriate.

In order to study a possible role of obesity on the FeT curves characteristics (peak force, area under the curve, and time to reach maximum plateau), the One way Analysis of Variance (ANOVA) test with Bonferroni post-hoc analysis has been applied, distinguishing subjects by categories of BMI (BMI ≤24.9 normal, BMI ≥25 and ≤29.9 overweight, BMI ≥30 obese).

The correlations between HGs curve characteristics and questionnaires were studied through the Spearman’s rho correlation coefficients (rho). Correlations >0.90 were interpreted as very high, 0.70–0.89 as high, 0.50–0.69 as moderate, 0.26–0.49 as low, and ≤0.25 as little if any correlation^22^. The HGs thresholds that best discriminates between the presence or the absence of FM, as well as between severe or not severe (not severe included modetate-mild-remission states) FM were determined using the receiver operating characteristic (ROC) curve analysis, applying the PASS as external criterion.

To identify the best HGs threshold, the closest point to (0, 1) of the ROC curve was chosen.

Finally, socio-demographic variables (age, BMI, level of education) and clinimetric parameters (WPI FIQR, sleep and fatigue), were entered as possible explanatory variables in the multivariate regression model, with HGs considered as dependent variable. The level of significance was set at P less than 0.05 for all the analyses.

## Results

### Demographic characteristics and descriptive statistics

One hundred and ten consecutive FM female patients were included. The mean age was 53.8 years (SD = 12.4, range: 18–80), with a mean time since the onset of chronic widespread pain of 5.6 years (SD = 4.3, range: 1–22). The mean body mass index (BMI) was 26.5 ± 2.2 kg/m^2^. Fewer than 25% of the patients were highly educated. The overall mean for FIQR was 52.6 (SD = 22.9), and for FAS was 6.1 (SD = 2.3). Matched female healthy subjects (111 controls) were comparable in terms of age (55.2 ± 14.9 years) and the mean BMI (25.9 ± 3.4). There was no significant difference in the distribution of age, gender marital status, and educational level between the two groups. The curve characteristis (peak force, time to reach maximum plateau, and area under the curve) in FM patients and healthy controls are described in Table 1.

**Table 1:** The force–time curves characteristics (peak force, time to reach maximum plateau, and area under the curve) in fibromyalgia patients and healthy controls.

HGs characteristics	FM (110 patients)				Healthy controls (111 subjects)			
	Mean	Median	SD	25–75 P	Mean	Median	SD	25–75 P
Peak Force (Kg/30 sec)	14.78	13.91	4.74	11.03–18.72	19.90	19.36	5.39	16.41–23.55
Time to reach maximum plateau (sec)	15.50	15.00	6.36	10.00–20.00	11.82	10.00	5.67	7.50–15.00
Area under the curve	363.79	342.76	115.47	267.66–464.96	501.84	496.55	137.86	410.12–585.26

Abbreviations: HGs = handgrip strength, FM = fibromyalgia, SD = standard deviation, P = percentile.

**Figure 2:**
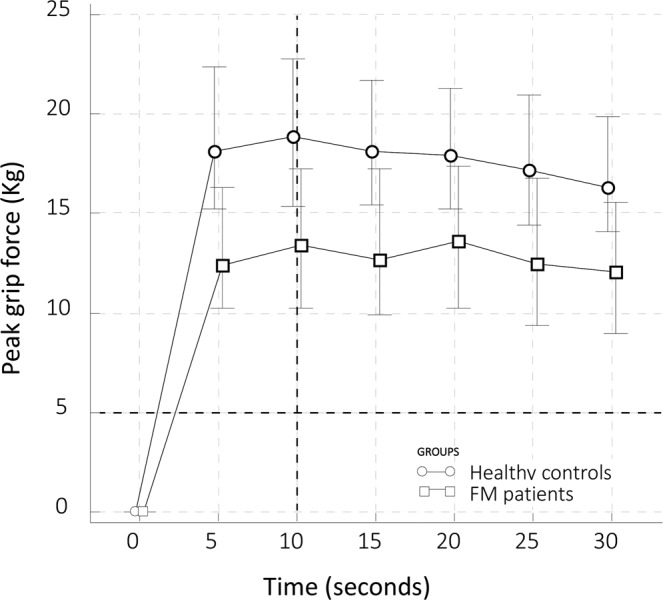
Median values (and 25–75 percentiles) of the handgrip strength peak force detected every five second during the 30 second of assessment in fibromyalgia syndrome patients and healthy controls.

Figure 2 depicts the median values of the peak force detected during the measurement interval in FM patients and healthy controls. HGs-AUC and peak force were lower in FM patients compared with healthy women (median values 342.7 vs 496.5 and 13.9 Kg vs 19.9 Kg, both p < 0.001), and in women with severe FM compared with those with not severe FM (Kruskal-Wallis test, p < 0.0001) (Tables 2 and 3). The time to reach the maximum plateau of the curves was significantly higher in patients with FM than healthy women (15.50 sec vs 11.82 sec, p < 0.001) (Table 1).

**Table 2:** Comparison of HGs peak force levels and HGs-AUCs for different disease severity states measured by FIQR.

FIQR vs HGs peak force levels	n	Minimum	25th percentile	Median	75th percentile	Maximum
High severity	39	6.36	9.52	11.51	13.68	20.57
Mild severity	11	9.54	14.61	18.52	21.39	25.84
Moderate severity	33	5.39	10.27	13.08	15.21	21.42
Remission	27	10.72	17.18	18.80	22.05	23.98
Test statistic	37.88					
Corrected for ties Ht	37.88					
Significance level	p < 0.0001					
**FIQR vs HGs-AUC**	**n**	**Minimum**	**25th percentile**	**Median**	**75th percentile**	**Maximum**
High severity	39	157.06	239.48	275.55	334.08	518.78
Mild severity	11	233.73	371.17	442.12	503.91	642.91
Moderate severity	33	134.58	248.36	324.96	384.77	537.55
Remission	27	258.70	422.57	468.11	525.58	594.30
Test statistic	37.62					
Corrected for ties Ht	37.62					
Significance level	p < 0.0001					

Abbreviations: HGs = handgrip strength, AUC = area under the curve, FIQR = revised Fibromyalgia Impact Questionnaire.

**Table 3:** Comparison of HGs peak force levels and HGs-AUCs for different disease severity states measured by FAS.

FAS vs HGs peak force levels	n	Minimum	25th percentile	Median	75th percentile	Maximum
High severity	52	5.39	9.51	11.97	13.95	21.53
Mild severity	24	8.21	13.62	16.85	18.96	22.14
Moderate severity	18	6.36	12.17	14.04	18.34	21.52
Remission	16	11.28	17.98	20.65	23.61	25.84
Test statistic	33.36					
Corrected for ties Ht	33.36					
Significance level	p < 0.0001					
**FAS vs HGs-AUC**	**n**	**Minimum**	**25th percentile**	**Median**	**75th percentile**	**Maximum**
High severity	52	134.58	234.56	285.26	342.76	546.12
Mild severity	24	210.42	350.31	426.91	472.55	511.22
Moderate severity	18	157.06	291.95	351.80	464.88	509.65
Remission	16	284.90	441.25	511.34	570.12	642.91
Test statistic	34.54					
Corrected for ties Ht	34.54					
Significance level	p < 0.0001					

Abbreviations: HGs=handgrip strength, AUC = area under the curve, FAS = Fibromyalgia Assessment Status.

ROC curve analysis revealed that the HGs peak force threshold that best discriminated between the presence or the absence of FM was 14.2 Kg (AUC 0.801; 95% confidence interval [CI] 0.723–0.844, p < 0.001), whereas the HGs peak force threshold that best discriminate the PASS was 16.3 kg (AUC 0.834; 95% CI. 0.751–0.898; p < 0.001).

As regards a potential role of obesity in FeT curves characteristics, investigated by distinguishing patients in three categories of BMI (Table 4), the ANOVA test did not allow to document significant differences of FeT curve characteristics between the different categories (Table 5).

**Table 4:** Comparison of force-time curves characteristics (peak force, area under the curve, and time to reach maximum plateau) in relation to BMI categories (BMI ≤24.9 normal, BMI ≥25 and ≤29.9 overweight, BMI ≥30 obese).

	BMI (Kg/m^2^)	N	Mean	Std. Deviation	Std. Error	95% Confidence Interval for Mean		Minimum	Maximum
						Lower Bound	Upper Bound		
HGs Peak Force (Kg/30 sec)	Normal	23	15.33	5.76	1.22	12.77	17.890	5.40	23.98
	Overweight	77	14.72	4.41	0.50	13.72	15.728	6.56	25.84
	Obese	10	14.20	5.37	1.70	10.36	18.051	6.36	21.10
	Total	110	14.78	4.74	0.45	13.89	15.684	5.40	25.84
HGs-AUC	Normal	23	378.87	1.41	30.16	316.14	441.601	134.58	594.30
	Overweight	77	360.87	1.06	12.12	336.73	385.010	163.36	642.91
	Obese	10	357.36	1.35	42.87	260.37	454.349	157.06	530.90
	Total	110	363.79	1.15	11.00	341.96	385.612	134.58	642.91
Time to reach the maximum plateau (sec)	Normal	23	14.32	5.83	1.24	11.73	16.90	5	25
	Overweight	77	16.23	7.35	0.83	14.57	17.90	5	30
	Obese	10	18.50	7.09	2.24	13.43	23.57	5	30
	Total	110	16.09	7.05	0.67	14.76	17.42	5	30

Abbreviations: HGs = handgrip strength; AUC = area under the curve; BMI = body mass index.

**Table 5:** One way Analysis of Variance (ANOVA) with Bonferroni post-hoc analysis of the role of BMI categories (BMI ≤24.9 normal, BMI ≥25 and ≤29.9 overweight, BMI ≥30 obese) in force-time curves characteristics (peak force, area under the curve, and time to reach maximum plateau).

		Sum of Squares	df	Mean Square	F	Sig.
HGs Peak Force (Kg/30 sec)	Between Groups	12.22	3	4.07	0.177	0.912
	Within Groups	2438.83	106	23.01		
	Total	2451.06	109			
HGs-AUC	Between Groups	7885.91	3	2628.64	0.193	0.901
	Within Groups	1.44	106	13636.93		
	Total	1.45	109			
Time to reach the maximum plateau (sec)	Between Groups	144.02	3	48.01	0.965	0.412
	Within Groups	5275.06	106	49.76		
	Total	5419.09	109			

Abbreviations: BMI = body mass index; HGs = handgrip strength; AUC = area under the curve.

In FM patients, a negative correlation was found between FIQR, FAS, widespread pain index (WPI) scores and peak force (all at p < 0.0001) and, furthermore, a correlation was observed between WPI and the time to reach maximum plateau of the curves (p = 0.041) (Table 6). No correlation was found between HGs and BMI in FM patients.

**Table 6:** Correlations between force-time curve characteristics and questionnaires studied through the Spearman’s rho correlation coefficients (rho).

	FIQR	FAS	HGs peak force levels	Time to reach maximum plateau of the curves	HGs-AUC
WPI	0.732 < 0.0001	0.823 < 0.0001	−0.612 < 0.0001	−0.195 0.0415	−0.615 < 0.0001
FIQR		0.761 < 0.0001	−0.576 < 0.0001	−0.054 0.5768	−0.592 < 0.0001
FAS			−0.577 < 0.0001	−0.167 0.0813	−0.588 < 0.0001
HGs peak force levels				−0.151 0.0249	0.991 < 0.0001
Time to reach maximum plateau of the curves					−0.135 0.0456

Abbreviations: HGs = handgrip strength, FIQR = revised Fibromyalgia Impact Questionnaire, FAS = Fibromyalgia Assessment Status, AUC = area under the curve, WPI = Widespread Pain Index.

Factors significantly associated with HGs-AUC in multivariate analysis were WPI (p = 0.003) and FIQR (p = 0.016) (Table 7).

**Table 7:** Multivariate logistic regression analysis of the variables contributing to HGs-AUC.

Independent variables	β coefficient	standard error	t	p	r_partial_	r_semipartial_
(Constant)	23.9781					
Age (years)	−0.03262	0.04084	−0.799	0.426	−0.08583	0.05902
BMI (Kg/m^2^)	−0.03895	0.09174	−0.425	0.672	−0.04573	0.03137
Level of education,yrs	−0.1127	0.1300	−0.867	0.388	−0.09305	0.06404
Disease duration,yrs	−0.02357	0.07027	−0.335	0.738	−0.03615	0.02479
WPI (0–19)	−0.6788	0.1850	−3.670	0.003	−0.3680	0.2712
FIQR (0–100)	−0.7892	0.2492	−3.167	0.016	−0.3231	0.2340
Sleep (NRS 0–10)	0.1213	0.2294	0.529	0.598	0.05694	0.03907
Fatigue (NRS 0–10)	0.2174	0.1463	1.486	0.141	0.1582	0.1098
R^2^ = 0.5183						
R^2^-adjusted = 0.4773						

Abbreviations: HGs = handgrip strength, AUC = area under the curve, BMI = body mass index, WPI = Widespread Pain Index, FIQR = revised Fibromyalgia Impact Questionnaire, NRS = numerical rating scale.

## Discussion

In this study we explored the characteristics of a new method of measuring HGs in FM patients based on FeT curve analysis, using a new device. From the studies available in the literature, there are several dynamometers available for measuring HGs, each with strengths and weaknesses. Among those most studied certainly can be recorded Jamar® and Takei®. The former has been used since the 50 s of the twentieth century^23^, and today is the one with the most abundant evidence^24,25^. Among the other instruments, the Takei® digital dynamometer has found a good diffusion in recent years^26^. Takei® is equipped with a digital display that measures the maximum force exerted by the flexor with a decimal figure and an adjustable handle that can be adapted to different amplitude measurements of the hand (from 3.5 to 7.0 cm). The main advantages of dynamometers such as Jamar® and Takei® are the reduced cost and practicality of use, while an important limit is represented by the possibility of evaluating only the isometric force and can cause stress on weak joints developing slow leaks and hysteresis. Compared to the anatomically shaped Jamar® handle, the Takei® handle has a straight profile that does not respect the natural posture of the fingers of the hand. The force evaluated by these two dynamometers is essentially unidirectional since the handle only allows a movement from the fingers to the proximal palm and thumb. This type of force measurement may not adequately represent the activities that the hand must perform to handle a cylindrical object. A work by McDowell and colleagues revealed how much more force is required to apply to a cylindrical object, dynamometers such as Jamar® and Takei® in particular incorrectly measure the force applied by the distal portion of the fingers^27^.

Taking all these considerations into account and to overcome the limitations of the currently available dynamomethers, we decided to develop a new cylindrical shape device, which would not only measure the peak force data as a result of the simple isometric muscle test, but a continuous acquisition of data each five second which lead to a more accurate information of the HGs of patients. The use of the FeT curve analysis improves the assessment of HGs, being able to evaluate magnitude and duration^12^. A sustained grip, as measured by HGs-AUC, is considered to reflect more closely the performance required in the execution of daily life activities compared to the peak grip force^28^. As far as we know, ours is the first study to use this methodology in the evaluation of HGs in FM patients.

Reduced physical performance, as measured by HGs, is not a new finding in FM^5,6,8,29,30^, and a research has also established physical fitness cut-offs for severity of illness^31^. Our results showed that HGs is reduced by about 30% in women with FM versus healthy controls. This reduction correlates with WPI and disease severity. Both HGs-AUC and peak force levels were associated with WPI, FIQR, and FAS scales, which are the instruments designated to assess pain, fatigue, activity limitations and mood.

The time to reach maximum plateau of the curves was predominantly related to the WPI. Low fitness and reduced exercise capacity have already been described in the context of FM. Compared to healthy women, muscle strength in women with FM is reduced in terms of 20–36%, while walking ability appears to be 16% lower^7^. Reduced endurance and easier fatigability have also been described, even for large muscle masses such as the quadriceps, but also for the abdominal and lumbar muscles^32–34^. Patients with FM have a lower maximum voluntary muscle strength, both isokinetic and isometric^35^. The causes of the altered muscle performance are to be found in the deconditioning that could result from fatigue, muscle damage, but also psychological variables^4^. In FM, reduced muscle strength was also related to pain^36^.

In healthy subjects, HGs is correlated to knee extension strength and can therefore be a surrogate for the strength of other muscle groups^37^. From this point of view, a relative simple measurement of the HGs can reflect a general assessment of muscle fitness in FM patients, therefore HGs can be a supportive tool to the clinical evaluation in establishing a treatment strategy. The HGs measurement can be particularly useful in the multimorbidity framework to detect patients with poor outcomes, such as the risk of hospitalization, which can benefit from close monitoring^38–40^.

The association between FM and reduced HGs has been demonstrated in different contexts^38,41,42^. Discordant data also exist, in which no associations between FM severity and reduced muscle performance, expressed in terms of isokinetic flexion-extension of the knee, has been demonstrated^43^.

Although somewhat controversial, the pathophysiological causes of the reduction in muscle strength should be sought in alterations of the muscle fibres themselves^44^, or in vasomotor dysregulation^45^, or in problems of energy metabolism and the regulation of growth factors^46^. Electrophysiological studies have also been conducted revealing the presence of functional alterations of the muscle membrane responsible for fatigue^47^.

Potential reasons also involve neuromuscular alterations caused by pain and decreased physical activity. Female FM patients usually dedicate more time to sedentary tasks rather than to activities requiring high muscle engagement. Pain and poor fitness cause physical inactivity which in turn reduces muscle conditioning^43^.

Our data did not reveal an HGs deficiency linked to the possible presence of overweight or obesity. This is consistent with what Carbonell-Baeza *et al*. have documented in a work in which they demonstrated an association of BMI with static balance, back scratch, and 6-minute walking test, but not with HGs^34^.

The present study has several limitations to mention. First of all, the cross-sectional assessment did not allow the evaluation of causal inferences (i.e., patients with higher disease severity may complain more pain and fear of movement, resulting in reduced strength). In addition and as a consequence, the cross-sectional evaluation did not allow prognostic evaluations (in terms of possible therapeutic interventions) of our new HGs measurement method. Finally, as men have not been studied, these findings are only applicable to the female population.

The device is currently not available on a large scale. However, considering the low production costs, we believe it will be widely available in the coming years.

In conclusion, confirming that HGs is reduced in patients with FM and that this correlates with the disease severity, this study proposes a new method (using a cylindrical-shape grip device) of evaluating HGs based on force-time curve analysis, trying to overcome some limitations of the dynamometers available today.
